# Pregnancies following long luteal phases in southern white rhinoceros (*Ceratotherium simum simum*)

**DOI:** 10.1002/zoo.21529

**Published:** 2019-12-03

**Authors:** Parker M. Pennington, Kira L. Marshall, Jonnie M. Capiro, Lauren Howard, Barbara S. Durrant

**Affiliations:** ^1^ San Diego Zoo Global Escondido California; ^2^ Animal Husbandry and Management San Diego Zoo Safari Park Escondido California; ^3^ Veterinary Services San Diego Zoo Safari Park Escondido California

**Keywords:** artificial insemination, assisted reproductive technology, estrous cycle, pregnancy, southern white rhinoceros

## Abstract

All extant species in the *Rhinocerotidae* family are experiencing escalating threats in the wild, making self‐sustaining captive populations essential genetic reservoirs for species survival. Assisted reproductive technologies (ARTs) will become increasingly important for achieving and maintaining ex situ population sustainability and genetic diversity. Previous reports have shown that a large proportion of captive southern white rhinoceros (SWR) females are irregularly cyclic or acyclic, and that cycling females display two different estrous cycle lengths of approximately 30 or 70 days. It has been suggested that the longer estrous cycle length is infertile or subfertile, as no term pregnancies have been observed following long cycles. Here we report the achievement of two pregnancies following long luteal phases, using ovulation induction and artificial insemination with either fresh or frozen‐thawed semen. One female SWR conceived on the first insemination attempt and gave birth to a live offspring. A second female conceived twice in consecutive long cycles although the first embryo was resorbed by 33 days post‐insemination. A pregnancy from this female's second insemination is ongoing with expected parturition in November 2019. Whether prolonged estrous cycles in SWR are subfertile or infertile in natural breeding situations remains unclear. However, our findings demonstrate that the application of ARTs following prolonged cycles can result the successful establishment of pregnancies in SWR. Therefore, with ARTs, female SWR otherwise considered nonreproductive due to long estrous cycles may still have the potential for representation and contribution to the ex situ population.

## Research Highlights

- Two pregnancies were achieved following long luteal phases in two southern white rhinoceros females.
- We provide evidence that long luteal phases are not necessarily infertile or early pregnancy loss.

## Graphical abstract

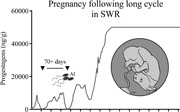

## 1 INTRODUCTION

Although the southern white rhinoceros (SWR) population is the most robust of the five species, demand for rhino horn remains high and poaching poses a significant threat to the wild population. Captive populations are approaching sustainable numbers, but the majority of females are characterized as acyclic or irregularly cyclic (Brown, Bellem, Fouraker, Wildt, & Roth, 2001; Hildebrandt et al., 2007; Roth, 2006; Roth, Schook, and Stoops, 2018), leaving many females unrepresented and limiting genetic diversity. Assisted reproductive technologies (ARTs) including artificial insemination (AI) could be useful tools to overcome the reproductive challenges faced by captive SWR and enhance genetic diversity.

The SWR exhibits two different estrous cycle lengths of approximately 30 or 70 days (Brown et al., 2001; Hindle, Möstl, & Hodges, 1992; Patton et al., 1999; Radcliffe, Czekala, & Osofsky, 1997; Schwarzenberger et al., 1998; Van der Goot, Martin, Millar, Paris, & Ganswindt, 2015; Roth, Schook, and Stoops, 2018). However, no mechanistic explanation for this difference has yet been identified. The longer cycle lengths are the result of prolonged luteal phases and have been described both ultrasonographically and hormonally (Brown et al., 2001; Patton et al., 1999; Pennington, Marshall, Capiro, Felton, & Durrant, 2019; Radcliffe et al., 1997; Schwarzenberger et al., 1998). Individuals may display one or both cycle lengths, but there is disagreement in the literature about whether long cycles are normal (Pennington et al., 2019; Schwarzenberger et al., 1998) or abnormal (Patton et al., 1999, Radcliffe et al., 1997) based on frequency and evidence of pathology. In addition, no full‐term pregnancies had been documented following long cycles, leading to the suggestion that long luteal phases are infertile or subfertile (Patton et al., 1999; Roth, 2006), although no direct scientific evidence supports this notion. Here we provide evidence to the contrary by documenting the first known established pregnancies following long luteal phases. For the purposes of this discussion, we define established pregnancies as those that develop beyond 160 days, or roughly one‐third of gestation. The combined use of ovulation induction and AI resulted in these pregnancies and provide a means for previously unrepresented females to contribute to the critical ex situ population.

## 2 METHODS

Three SWR females (SB#s 2194, 2197, 2198) aged 8–10 years (estimated) were trained for routine ultrasound exams without sedation during which ovarian characteristics and structures were documented. When follicles reached preovulatory size (>30 mm diameter), females received an ovulation induction treatment protocol (Pennington et al., 2019) with a gonadotropin releasing hormone analog (GnRH; SucroMate™, Bioniche Animal Health). Females were housed as a bachelorette herd with no opportunity to breed naturally. Fecal progesterone metabolites were monitored by radioimmunoassay for each female, as previously described (Pennington et al., 2019).

Two male SWR (SB#s 1081, 1241) contributed semen for seven AIs. Under a surgical plane of anesthesia semen was collected by electroejaculation using a specially designed probe (Roth et al., 2005). Semen was collected twice from male #1241 and cryopreserved in liquid N_2_ in either BotuCrio® (Botupharma, Brazil) or CryoMax™ (ARS Inc.). Semen from both ejaculates was thawed, centrifuged, pooled, and resuspended in INRA96® (IMV Technologies, France) before inseminating female #2197. A single semen collection from male #1081 was extended in INRA96®, chilled overnight at 4°C, then warmed to room temperature before inseminating female #2194. Each insemination dose that resulted in conception contained at least 500 × 10^6^ motile sperm.

AIs were conducted in a chute, under a butorphanol (20–40 mcg/kg) and azaperone (20–40 mcg/kg) based standing sedation. A rigid stainless‐steel catheter was passed through the cervix to deposit sperm into the uterine body. Females were then given the reversal agent Naltrexone, and released for observation.

## 3 RESULTS

Seven insemination attempts in three females using this ovulation induction protocol resulted in three successful fertilizations in two females. Two of these fertilizations developed into established pregnancies. Female #2194 was inseminated four times and conceived twice. A conceptus was visualized 20 days following AI in April of 2018, but was no longer visible by Day 33. The AI on the subsequent ovulation was successful and at the time of this manuscript preparation is ongoing at >400 days. Female #2197 was inseminated once and her pregnancy concluded successfully after 493 days. A third female, #2198, was inseminated twice, both following long cycles (data not shown), without subsequent pregnancy.

**Figure 1:**
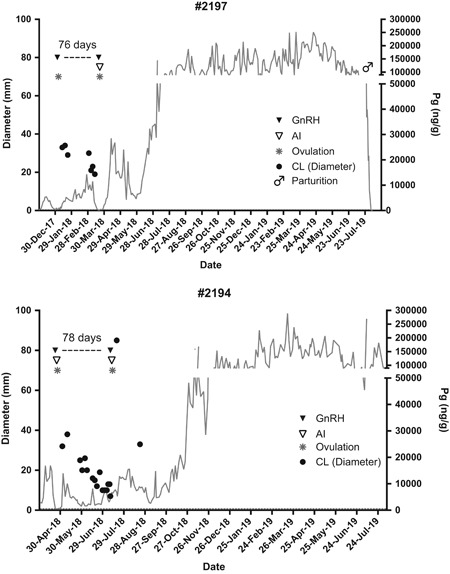
Progestagen profile for each pregnant female outlining GnRH treatments (▼) and time between. Closed circles (●) indicate corpus luteum visibility via ultrasound. Open triangles (∇) indicate AI procedures. Asterisks (*) indicate confirmed ovulation determined by ultrasound. Both progestogen elevation and corpus luteum visibility indicate long luteal phases before ovulations that resulted in ongoing pregnancies. AI, artificial insemination; CL, corpora lutea; GnRH, gonadotropin releasing hormone

The inter‐treatment interval (GnRH treatment to GnRH treatment) was 76 and 78 days and corpora lutea (CL) were visible for 60 and 77 days before GnRH treatment for females #2197 and #2194, respectively (Table 1, Figure 1). Inseminations that resulted in established pregnancies occurred in March and July of 2018 and pregnancies were diagnosed by ultrasound on Days 18 and 20 post‐ovulation for females #2197 and #2194, respectively. Viable fetuses are regularly observed by ultrasound. The male calf was born to female #2197 represents the first SWR calf born from AI in North America and the second ever from frozen‐thawed sperm (Hermes et al., 2009).

**Table 1:** Insemination parameters and time between GnRH treatments to induce ovulation for established pregnancies

Female (SB#)	Male (SB#)	Sperm preparation	GnRH 1	GnRH 2 (AI)	Interval (days)	CL visibility (days)	Expected calving
2197	1241	Frozen‐thawed	January 3, 18	20 March 18	76	60	July19^a^
2194	1081	Fresh, chilled	April 23, 18	10 July 18	78	77	November 19

Abbreviations: AI, artificial insemination; CL, corpora lutea; GnRH, gonadotropin releasing hormone

^a^ Healthy male calf born July 28, 2019.

## 4 DISCUSSION

The mechanism or possible adaptive significance of the two distinct estrous cycle lengths in SWR females is unknown. To our knowledge, SWR is the only species known to exhibit two distinct cycle types regularly. Ovulations following long luteal phases have been considered infertile or subfertile as they have not been associated with established pregnancies thus far and have been suggested to be caused by reproductive pathology or failed pregnancy (Patton et al., 1999; Radcliffe et al., 1997; Roth, 2006). However, more recent studies utilizing longitudinal ultrasound exams with long‐term hormone monitoring have observed long cycles in females without pathology or embryo loss (Hermes et al., 2009; Pennington et al., 2019). Although early embryo loss may be associated or concurrent with some observed long cycles, we demonstrate here that long cycles are not caused by embryo loss. Furthermore, the establishment of a pregnancy in female #2194 in a cycle immediately following a luteal phase in which early embryo loss occurred suggests that embryo loss does not impact the fertility of the subsequent cycle. To be certain, however, the successful establishment of more pregnancies following early embryo loss must be documented. Nonetheless, we offer that long luteal phases are not pathological or necessarily the result of embryonic loss (Pennington et al., 2019) and are not infertile.

The efficiency of AI in SWR is not yet known as the technique is still in development. AI is not consistently employed in this species and only three pregnancies have been described in the literature (Hildebrandt et al., 2007; Hermes et al., 2009). Here we report a total of seven AI attempts following long cycles, the detection of three conceptuses, and the establishment of two pregnancies. The birth of a healthy calf after 493 days of gestation in female #2197 indicates that these pregnancies, once established can be carried to term successfully. AI failures may be attributed to several factors including location of semen placement in the female's reproductive tract and sperm viability. As the application of ARTs to SWR becomes more effective, the establishment of pregnancies following long cycles offers even greater potential to rescue the reproductive potential of females that tend to display only long cycle types.

As our understanding of this species' reproductive physiology grows, previous perceptions of long cycle infertility may be amended. Though it is unclear if short and long cycles are equally fertile, the work presented here demonstrates that long cycles are not infertile. Therefore, the reproductive potential of females that display long cycles should not be discounted. Indeed, the failed AI attempts reported here may support the belief that long cycles are subfertile compared to short cycles. Additionally, cycle manipulation like GnRH treatment to induce ovulation, may be helpful in achieving established pregnancies by improving oocyte quality before ovulation. Work in cattle found that GnRH treatment before ovum pickup resulted in significantly better oocyte maturation rates and development to the blastocyst stage compared to untreated animals (Bordignon, Morin, Durocher, Bousquet, & Smtih, 1997; Ogata, Yu, Hidaka, Matzushige, & Maeda, 2016). However, pregnancy rates did not differ after transfer of either control or GnRH‐stimulated embryos (Ogata et al., 2016). In light of these studies, it is interesting to speculate that if longer luteal phase limits the developmental potential of oocytes ovulated at the following estrus, perhaps GnRH‐induced ovulation could promote oocyte quality, enhancing embryo developmental capacity and increasing the likelihood of establishing pregnancy. Therefore, SWR that display predominantly long cycles may benefit from GnRH treatment and exhibit greater potential for an established pregnancy. This approach could improve the genetic health of the SWR population by supporting reproduction in individuals that may have been presumed infertile.

During the preparation of this manuscript, a healthy female calf was born to female 2194 after 498 days of gestation.
